# Sample introduction valve used in ICP-AES for faster analysis

**DOI:** 10.1155/S1463924689000179

**Published:** 1989

**Authors:** Helge Stray

**Affiliations:** Agricultural Service Laboratory Box 91 1432 Ås-NLH Norway

## Abstract

A twin six-port valve with two sample loops was installed between the autosampler and the nebuliser of a simultaneous inductively coupled plasma-atomic emission spectrometer. The valve was mounted close to the nebuliser inlet so that the time required for the sample to enter from the loop to the nebulizer was less than 0.5 s. The content of one loop was introduced to the nebulizer using a peristalic pump, whilst a second loop was filed with the next sample using a second peristaltic pump. The washout time was in this manner reduced by 20 s per analysis and the hourly sampling rate was increased from 90 to 180 in the measurements described.


Journal of Automatic Chemistry Vol. 11, No. 2 (March–April 1989), pp. 84–86

### Sample introduction valve used in ICP-AES for faster analysis

**Helge Stray**
*Agricultural Service Laboratory, Box 91, 1432 Ås-NLH, Norway*

*A twin six-port valve with two sample loops was installed between the autosampler and the nebuliser of a simultaneous inductively coupled plasma-atomic emission spectrometer. The valve was mounted close to the nebuliser inlet so that the time required for the sample to enter from the loop to the nebulizer was less than 0·5 s. The content of one loop was introduced to the nebulizer using a peristaltic pump, whilst a second loop was filled with the next sample using a second peristaltic pump. The washout time was in this manner reduced by 20 s per analysis and the hourly sampling rate was increased from 90 to 180 in the measurements described.*

### Introduction

The sample introduction system most commonly used in inductively coupled plasma-atomic emission spectroscopy (ICP-AES) is a nebulizer similar to that used in flame atomic absorption spectroscopy (AAS). Optimum conditions for sample introduction, however, are quite different for the two analytical techniques. In ICP-AES the gas and liquid flow rates are approximately 1 l/min and 1 ml/min, respectively while the corresponding flow rates in AAS are typically 18 l/min and 6–8 ml/min [1]. The lower nebulizer flow rates in ICP-AES make the washout times for the sample introduction tube and the nebulizer substantially longer than with AAS. The sample is normally introduced to the ICP-nebulizer via a peristaltic pump making the necessary washout period even longer. The slower washout for ICP-AES sample introduction systems is usually the main reason for the rather low sampling rate encountered in simultaneous ICP-AES compared to AAS.

This article describes a valve system mounted close to the nebulizer inlet. While the first sample is in the plasma, the next is brought to the nebulizer and is ready to be sprayed into the nebulizer chamber immediately after the exposure period is terminated. In this way the sampling rate is increased and approaches the level achieved in AAS.

### Instrumentation

| | |
|---|---|
| Plasma instrument: | AUTOCOMP 1100 with nitrogen purged optical path. (Thermo Jarrell Ash, MA 02254, USA.) |
| Nebulizer: | Fixed cross flow. Cat. No. 90790. |
| Computer: | DEC MICRO-11 (PDP-11) with DEC MICRO/RSX operating system. |
| Autosampler: | Gilson Model 222. (Gilson Medical Electronics, Inc., WI 53562, USA.) |
| Valve actuator: | PSA 40·630 with twin 6-port Teflon valve. (P. S. Analytical Ltd, Kent BR5 3TR, UK.) |
| Peristaltic pump 1: | Cat. No. 39–601, single channel. (Rainin Instrument Co. Inc., CA 94608, USA.) |
| Peristaltic pump 2: | Masterflex pump. Cat. No. J-7554-50 with quick load head and tube size 14. (Cole-Parmer Instrument Co. Ltd, IL 60648, USA.) |
| In-line filter: | P/N 35331 with 35 micron filter P/N 36507. (DIONEX Corp., CA, USA.) |

The Teflon valve with the pneumatic actuator was dismantled from the box and fixed to the plasma instrument beside the nebulizer inlet. All internal flow paths of the Teflon valve, except the outlet leading to the nebulizer, were drilled to 1 mm diameter in order to reduce flow resistance.

The computer command file for the operation of the autosampler and the spectrometer was altered so that the autosampler was always one sample ahead of the one being analysed by the spectrometer.

The valve actuator was triggered from one of the slave relays at the rear of the spectrometer. A 'flip-flop' relay was installed between the slave relay and the actuator.

### Description of the sample valve

The sample valve and the liquid pathways are shown schematically in figure 1. Each sample loop is made up of

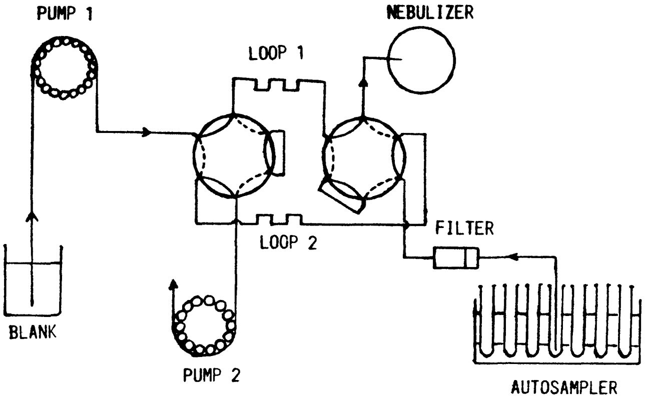

*Figure 1. Schematic presentation of sample introduction device with twin 6-port valve.*

0142–0453/89 $3.00 © 1989 Taylor & Francis Ltd.

a Teflon tube of internal diameter 1·5 mm and with an internal volume of 1·5 ml. When the valve is operated, the content of Loop 1 is pumped to the nebulizer by peristaltic pump 1. At the same time the autosampler moves to the next sample cup and the next sample is sucked into Loop 2 by peristaltic Pump 2 working at a flow rate of 15 ml/min. The tube from the valve to the nebulizer has an internal diameter of 0·3 mm and is 10 cm long. Since the flow rate of Pump 1 is adjusted to 2·4 ml/min, it takes less than 0·5 s for the sample to reach the nebulizer after the valve is operated.

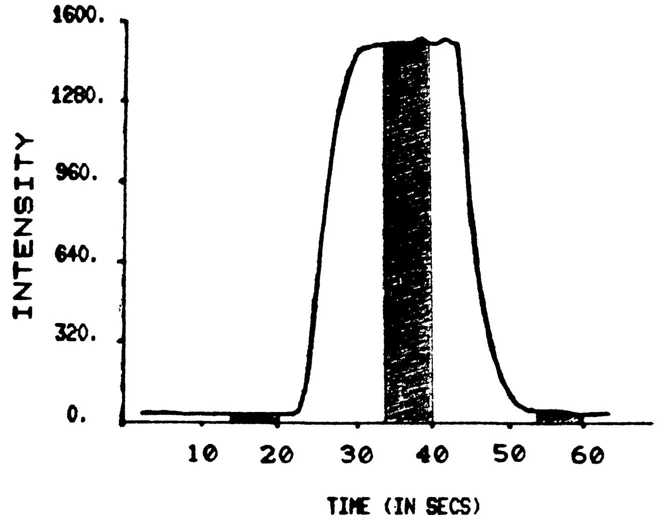

*Figure 2. Time study of intensity from 383·2 nm Mg-line. A blank, a standard (50 ppm), and a blank exposed successively using the valve system. Exposure periods applied in analysis of soil extracts cross-hatched.*

The reservoir for Pump 1 was filled with blank solution. The sample introduction valve could also have been made from a single 8-port Teflon valve, but no such valve was available on the market.

A volume of approximately 3 ml was found sufficient for flushing the filter, the loop, and the tubes with the sample solution.

### Results and discussion

The valve system was used for the analysis of P, K, Mg, Ca and Na in soil samples extracted by ammonium lactate solution. One ppm Li was added to the extracting solution and the concentration of Li in the extracts was measured along with the other elements. The soils analysed did not contain any significant amount of extractable Li, and the Li-content added could, therefore, be used for checking the performance of the sample introduction system.

The exposure time for the spectrometer was set to 6 s and the delay time of the autosampler also to 6 s. The remaining 8 s of the 20 s analysis time was used by the autosampler to move to the next sample cup (7 s) and for computer operations. An exposure time of 6 s offered sufficient sensitivity for the analysis. Background correction was not found necessary.

*Table 1. Wavelengths applied, detection limit and precision achieved for the analysis.*

| Element | Wavelength (nm) | Detection limit (mg/l) | Concentration (mg/l) | Precision (% RSD) |
|---|---|---|---|---|
| P | 177·4 | 0·04 | 25·0 | 0·4 |
| K | 766·4 | 0·28 | 56·5 | 0·8 |
| Mg | 383·2 | 0·06 | 50·0 | 0·5 |
| Ca | 317·9 | 0·17 | 500·0 | 1·4 |
| Na | 588·9 | 0·21 | 25·0 | 0·5 |
| Li | 670·7 | – | 1·0 | – |

*Table 2. Mean percentage carry-over analysing a standard and blank successively 10 times.*

| Element | Concentration (mg/l) | Mean carry-over (mg/l) | Carry-over (%) |
|---|---|---|---|
| P | 25 | 0·11 | 0·44 |
| K | 56·5 | 0·25 | 0·44 |
| Mg | 50 | 0·23 | 0·46 |
| Ca | 500 | 1·92 | 0·38 |
| Na | 25 | 0·10 | 0·40 |

Six hundred samples were measured each day. The blank and the standard solution were checked after every 25 samples and a restandardization was automatically performed after each 75 samples.

Figure 2 shows a time study of the intensity from the 383·2 nm Mg-line when a blank, a standard solution and a blank were introduced into the plasma successively using the sample value. The exposure periods of the spectrometer applied for the analysis of soil extracts are cross-hatched in the figure.

On analysing 10 standard solutions successively the percentage relative standard deviation ranged from 0·4 for P to 1·4 for Ca (table 1). The precision was similar to that reported by other users of the same instrument with traditional sample introduction using 10 s exposure time [2]. A somewhat better precision obtained using the valve system can be explained by less instrument drift during the shorter analysis time. In addition, no air is entering the nebulizer during the analysis sequence and this may be advantageous in terms of the stability of the plasma.

Table 2 shows the mean values obtained after a standard and a blank were analysed successively 10 times. The standard solution contained 25, 56·5, 50, 500, and 50 mg/l of P, K, Mg, Ca and Na respectively. As can be seen from table 2, the mean carry-over was 0·46% or less for all elements (see also figure 2). The carry-over is due to some standard solution still remaining in the nebulizer, not in the sample valve, and can be reduced by prolonging the autosampler delay time. The concentration of the measured elements would normally range from 1 to 1000 and correction for the carry-over was only necessary in special cases. The valve system has also been used with other analytical programs where background correction was used for the analysis of 20 elements with a total exposure time of 20 s. Because of the valve system one analysis could still be performed in 36 s. The flow rate of Pump 2 was then reduced to 6 ml/min. If the valve system is to be used for programs with exposure times exceeding 20 s, larger loops must be installed.

### References

1. Willard, H. H., Merritt, L. L., Dean, J. A. and Settle, F. A., *Instrumental Methods of Analysis* (Wadsworth Publishing Company, Belmont, CA, 1988).
2. Holmberg, M., *Kemia-Kemi* (Finland) No. 6 (1986), 554–556.

### Calendar

### MARCH

6–10 March 1989 *Pittsburgh Conference 1989: Atlanta, GA, USA.* Contact Pittsburgh Conference, Suite 322, 12 Federal Drive, Pittsburgh, PA 15235, USA.

7–9 March 1989 *Workshop: Statistics for Analytical Chemists: Eastbourne, UK.* Contact Christine Robinson, Statistics for Industry (UK) Ltd, 4 Victoria Avenue, Knaresborough, North Yorks HGF 9EU, UK.

21–22 March 1989 *Research and Development Topics in Analytical Chemistry: Dublin, Ireland.* Contact Analytical Division, Royal Society of Chemistry, Burlington House, Piccadilly, London W1V 0BN.

30 March 1989 *Analytical Atomic Spectroscopy in the Environment: Glasgow, UK.* Contact Analytical Division, Royal Society of Chemistry, Burlington House, Piccadilly, London W1V 0BN.

### APRIL

4–7 April 1989 *RSC Annual Congress: Hull, UK.* Contact Gina B. Howlett, Conference Officer, RSC, Burlington House, Piccadilly, London W1V 0BN. The Analytical Division session will be on *Process Analysis and Information Management.*

5–6 April 1989 *Laboratory Science and Technology Show: Cambridge, UK.* Contact Curtis Steadman and Partners Ltd., The Hub, Emson Close, Saffron Walden, Essex CB10 1HL, UK.

11–14 April 1989 *VIIth International Symposium on Electroanalysis and Sensors in Biomedical, Environmental and Industrial Sciences: Loughborough, UK.* Contact Dr A. G. Fogg, Chemistry Department, University of Loughborough, Leicestershire LE11 3TU, UK.

12–13 April 1989 *Laboratory Manchester: Manchester, UK.* Contact Curtis Steadman & Partners Ltd., The Hub, Emson Close, Saffron Walden, Essex CB10 1HL, UK.

19 April 1989 *Chemometric Techniques in Clinical Chemistry and the Pharmaceutical Industry: Salford, UK.* Contact Analytical Division, Royal Society of Chemistry, Burlington House, Piccadilly, London W1V 0BN.

### JUNE

25–30 June 1989 *Eurosensors III and 5th International Conference on Sensors and Actuators: Montreux, Switzerland.* Contact Eurosensors (Transducers '89), COMST S.A., PO Box 415, 1001 Lausanne 1, Switzerland.

### JULY

30 July–5 August 1989 *SAC 89: Cambridge, UK.* Contact Analytical Division, Royal Society of Chemistry, Burlington House, Piccadilly, London W1V 0BN.

### SEPTEMBER

11–13 September 1989 *RSC/SCI/IOP Analysis Techniques and Applications: Manchester, UK.* Contact Mrs E. S. Wellingham, Field End House, Bude Close, Nailsea, Bristol BS19 2FQ, UK.

25–27 September 1989 *Sensors and their Applications IV: Canterbury, UK.* The Meetings Officer, Institute of Physics, 47 Belgrave Square, London SW1X 8QX.

25–28 September 1989 *Third International Symposium on Kinetics in Analytical Chemistry: Dubrovnik, Yugoslavia.* Contact Professor Gordana A. Milonović. Department of Chemistry, University of Belgrade, KAC PO Box 550, 11001 Belgrade, Yugoslavia.

26–28 September 1989 *The British Laboratory Week: Olympia, London.* Including Laboratory 89, Computer Aided Sciences, Bio 89, Medical Laboratory Sciences and Analyticon. Contact Curtis Steadman and Partners, The Hub, Emson Close, Saffron Walden, Essex CB10 1HL.

